# Castration‐resistant prostate cancer diagnosed during leuprorelin treatment for spinal and bulbar muscular atrophy

**DOI:** 10.1002/iju5.12447

**Published:** 2022-04-10

**Authors:** Atsuhi Yanase, Toru Sugihara, Takahiro Akimoto, Hirotaka Yokoyama, Jun Kamei, Akira Fujisaki, Satoshi Ando, Tameto Naoi, Mitsuya Morita, Tetsuya Fujimura

**Affiliations:** ^1^ Department of Urology Jichi Medical University Shimotsuke Japan; ^2^ Rehabilitation Center Jichi Medical University Shimotsuke Japan

**Keywords:** androgen receptor, leuprorelin acetate, motor neuron disease, prostate cancer, spinal and bulbar muscular atrophy

## Abstract

**Introduction:**

We report a prostate cancer case diagnosed during leuprorelin treatment for spinal and bulbar muscular atrophy which is a X‐linked recessive, lower motor neuron disease.

**Case presentation:**

A 64‐year‐old man who had received leuprorelin treatment over 3 years for his spinal and bulbar muscular atrophy presented with an enlarged prostate accompanied by abdominal pain and constipation. An abnormally high serum prostate‐specific antigen of 17.7 ng/mL and a low (castration level) serum testosterone level of 0.23 ng/mL were measured. Prostate needle biopsy revealed adenocarcinoma of the prostate. Orchiectomy, darolutamide, and radiation therapy for the prostate were initiated, resulting in a favorable response which was maintained at 12 months of treatment.

**Conclusion:**

Prostate cancer can occur even when leuprorelin is used for spinal and bulbar muscular atrophy; therefore, checking serum prostate‐specific antigen to screen for prostate cancer before leuprorelin administration should be considered.

**Abbreviations & Acronyms**
ADT: androgen deprivation therapy
AR: androgen receptor
CT: computed tomography
IMRT: intensity modulated radiation therapy
PSA: prostate‐specific antigen
SBMA: spinal and bulbar muscular atrophy

**Keynote message**

Prostate cancer can occur even when leuprorelin is used for spinal and bulbar muscular atrophy. Leuprorelin masks serum PSA levels. Therefore, there was room for consideration of serum PSA evaluation prior to leuprorelin administration to men with spinal and bulbar muscular atrophy.

## Introduction

SBMA, otherwise known as Kennedy's disease, is a rare X‐linked recessive, lower motor neuron disease, caused by a cytosine‐adenine‐guanine (CAG) trinucleotide repeat expansion in the first exon of the AR gene.^1^, ^2^, ^3^ In Japan, leuprorelin acetate is approved to inhibit the progression of this disease. We report here a 64‐year‐old man who was diagnosed with prostate cancer during leuprorelin treatment for SBMA.

## Case presentation

A 64‐year‐old man was referred to our hospital with irregularly enlarged prostate; this was found during an examination in which his chief complaints were of abdominal pain and constipation. He had already been administrated leuprorelin acetate as a treatment for SBMA for the past 3 years.

The patient had been aware of arm tremors since age 35, with gradual onset of dysarthria and dysphagia. At the age of 54, a genetic test revealed that the number of CAG repeats in his AR gene was abnormally long (46), and he had been diagnosed with SBMA. His older brother had also been diagnosed with SBMA. At age 55, the patient participated in a randomized controlled trial of leuprorelin acetate treatment, however, he was assigned to the placebo group. At the age of 60, he began receiving the actual drug, and his neurological symptoms stopped progressing thereafter. His serum PSA had never been measured at this point.

**Fig. 1:**
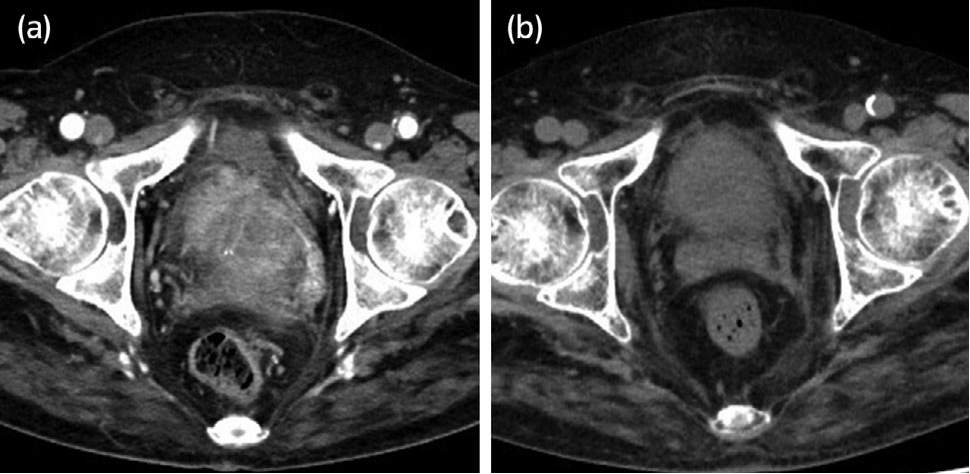
Pelvic CT scans at diagnosis (a) and after initiation of treatment for prostate cancer (b). (a) The contrast‐enhanced CT scan at the diagnosis revealed that the prostate was irregularly enlarged with a diameter of 84 mm, suggesting left seminal vesicle invasion of prostate cancer accompanied by a swelling right obturatorius lymph node. (b) The simple CT scan showed a significant decrease in size of prostate cancer after initiation of dalolutamide intake and radiation therapy to the prostate.

**Fig. 2:**
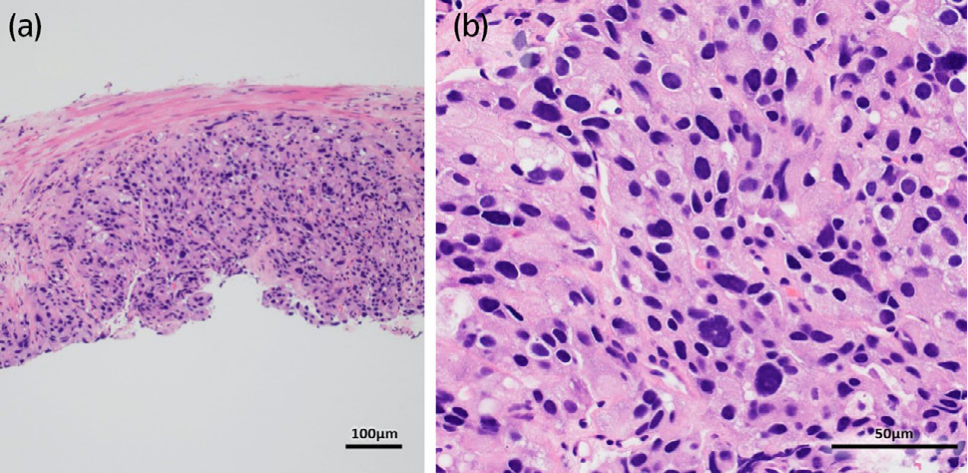
Hematoxylin–eosin staining of the prostate biopsy. Adenocarcinoma with collapsed ductal structure is observed. Gleason score cannot be determined because of post‐androgen deprivation therapy. (a) 10×. (b) 40×. [Colour figure can be viewed at wileyonlinelibrary.com]

After the visit to our hospital, a laboratory test revealed that his serum PSA level was abnormally high at 17.7 ng/mL, even though his serum testosterone level had dropped to the castration level of 0.23 ng/mL (reference value, 1.31–8.71 ng/mL). Contrast‐enhanced CT scan showed that his prostate was irregularly enlarged with a diameter of 84 mm, suggesting left seminal vesicle invasion of prostate cancer accompanied by a swelling right obturatorius lymph node (Fig. 1a). No evidence of distant metastasis was detected by CT scan and bone scintigraphy. Whole‐body MRI was not performed. Based on the clinical history, the patient was diagnosed with castration‐resistant prostate cancer without distant metastasis (m0CRPC). The patient underwent prostate needle biopsy and bilateral orchiectomies simultaneously, and the pathological results showed adenocarcinoma of the prostate (Fig. 2). The Gleason score could not be determined because the patient had already received leuprorelin acetate for several years. For treating m0CRPC, dalolutamide intake and radiation therapy to the prostate were initiated, resulting in a significant decrease in serum PSA levels and reduction in the size of the prostrate (Fig. 1b). This favorable response was maintained at least 12 months of treatment.

## Discussion

SBMA, also known as Kennedy's disease, is an adult‐onset, X‐linked recessive, lower motor neuron disease, characterized by an abnormal expansion of a CAG triplet repeat in the first exon of the AR gene. In healthy individuals, the number of CAG repeats in the AR gene ranges usually between 9–36, while patients with SBMA have between 38–62 CAG repeats.^1^, ^2^, ^3^ SBMA affects only males, and its incidence is 1–2 per 100 000 males.^2^ Abnormal accumulation of mutant AR proteins in the nuclei of motor neurons leads to slowly progressive limb muscle paralysis, muscle atrophy, and ball paralysis. A randomized controlled trial, JASMITT study, was executed to evaluate the efficacy of a leuprorelin acetate 11.25 mg injection in inhibiting the progression of the disease.^2^, ^3^ Based on the results of the JASMITT study, leuprorelin acetate 11.25 mg injection was approved for SBMA treatment in 2017 in Japan.

In the general population, several reports mentioned the association between the number of CAG repeats and prostate cancer. An inverse relationship between the number of repeats and the risk of developing prostate cancer was suggested; the shorter the number of CAG repeats, the more likely a man is to develop prostate cancer.^4^, ^5^ It is also well known that there are racial/ethnic differences in the incidence and prognosis of prostate cancer,^6^ and there is a theory that the variation in the number of CAG repeats by race/ethnicity could affect the racial/ethnic differences in prostate cancer profiles. Irvine *et al*.^7^ reported that those with CAG repeats of less than 22 were found in 75% of African Americans at high risk of prostate cancer, in 62% of non‐Hispanic whites at intermediate risk, and 49% of Chinese and Japanese at low risk. It has also been suggested that males with shorter CAG repeats tend to be diagnosed with prostate cancer at a younger age with a worse grade.^8^ Conversely, males with prostate cancer with longer CAG repeats tend to respond better to androgen deprivation therapy.^8^

This evidence suggests that SBMA patients with abnormally long CAG repeats are less likely to develop prostate cancer. To corroborate the evidence, a literature search for prostate cancer development among SBMA patients in PubMed (MEDLINE) and Ichushi‐web (Japanese medical literature) revealed that only six cases, including our case, had been reported (Table 1). Moreover, other than our case, no report was found that mentioned prostate cancer diagnosis during preceding leuprorelin treatment for SBMA. In two of these cases, germline *BRCA2* mutations with neuroendocrine differentiation pathology were identified. Considering that germline *BRCA2* variants are found in only 1.1% of Japanese prostate cancer patients, this is a very high frequency.^15^ Hongo *et al*.^13^ suggested that genomic instability caused by *BRCA2* mutations could cause androgen‐independent prostate cancer clones. In this case, we plan to perform cancer genomic profiling testing when distant metastases appear in the future.

**Table 1:** Previously reported cases of prostate cancer development among males with SBMA

No.	Author	Year	Age	PSA at diagnosis (ng/mL)	Treatment for prostate cancer	The number of CAG repeat	Complement
1	Yasui *et al*.^9^	1999	63	72.0	Fosphestrol	52	
2	Yoshida *et al*.^10^	2002	75	97.5	Orchiectomy with bicalutamide	n/a	Died of prostate cancer after 15 months
3	Sugahara *et al*.^11^	2011	66	n/a	Radical prostatectomy	n/a	
4	Kosaka *et al*.^12^ Hongo *et al*.^13^	2012	54	148.0	(i) ADT with flutamide (ii) Enzalutamide (iii) Carboplatin and etoposide	46	Survival at 16 years *BRCA2* mutation
5	Conteduca *et al*.^14^	2018	57	53	(i) ADT with docetaxel (ii) Enzalutamide (iii) Talazoparib	49	Survival at 31 years *BRCA2* mutation
6	Our case	2021	64	17.7	Orchiectomy with darolutamide and IMRT	46	Survival at 12 months Preceding leuprorelin use for SBMA

In this case, at the first time of oncological diagnosis, the prostate cancer was already castration‐resistant due to androgen deprivation therapy for SBMA. The exact timing of prostate cancer development is unknown due to the lack of previous PSA measurements. As the use of leuprorelin in patients with SBMA masks PSA levels, there was room for consideration of serum PSA evaluation prior to leuprorelin administration.

## Conclusion

We had the rare experience of diagnosing castration‐resistant prostate cancer in a man receiving leuprorelin for the treatment of SBMA. This case showed that prostate cancer can occur even when leuprorelin is used for SBMA, and that prostate cancer screening by checking serum PSA before leuprorelin administration should be considered.

## Author Contributions

Atsuhi Yanase: Writing – original draft. Toru Sugihara: Conceptualization; writing – original draft. Takahiro Akimoto: Writing – review and editing. Hirotaka Yokoyama: Writing – review and editing. Jun Kamei: Writing – review and editing. Akira Fujisaki: Writing – review and editing. Satoshi Ando: Writing – review and editing. Tameto Naoi: Conceptualization; supervision. Mitsuya Morita: Conceptualization; supervision. Tetsuya Fujimura: Supervision.

## Conflict of interest

The authors declare no conflict of interest.

## Approval of the research protocol by an Institutional Reviewer Board

The protocol for this research project was approved by a suitably constituted Institutional Reviewer Board at Jichi Medical University Hospital (Approval number, A19‐199).

## Informed consent

Informed consent was obtained from the subject.

## Registry and the Registration No. of the study/trial

Not applicable.
